# Appraisal of RCT on female hernia repair: methodological and clinical considerations

**DOI:** 10.1097/MS9.0000000000003949

**Published:** 2025-09-23

**Authors:** Kinza Siddique, Haseeb Safdar Ali, Muddassir Khalid, Supoma Ghosh Ria

**Affiliations:** a Department of Medicine, Nishtar Medical University, Multan, Pakistan; b Mugda Medical Coollege, University of Dhaka, Bangladesh

*To the Editor:*

We commend Matovu *et al* for their randomized trial, “Comparison of Modified Versus Standard Open Anterior Mesh Repair for Female Inguinal Hernia,” which addresses a critical gap in sex-specific surgical care. The authors’ modified technique, designed to improve femoral canal exposure, is particularly relevant given the high proportion of femoral hernias in their cohort (45%)[1]. The finding that this approach is safe, feasible, and shows a trend toward reduced chronic pain marks meaningful progress in tailoring care to female anatomy. However, several limitations warrant attention. Most notably, 35.4% of patients initially randomized to the standard repair group ultimately received the modified technique due to intraoperative detection of femoral hernias. While this decision was clinically appropriate, such a high crossover rate weakens the strength of the intention-to-treat analysis. The primary outcome of recurrence did not reach statistical significance (difference, − 3.0%; 95% CI, − 9.5% to + 3.4%; *P* = 0.36), yet the authors describe the modified repair as a good option. This may overstate the evidence, as the high crossover rate likely diluted any real differences between groups and biased the results toward no effect[2].

Additionally, the study lacks essential reporting on mesh specifications. Although the authors mention using a lightweight polypropylene mesh, they do not include important details such as pore size, weight, fixation method, or manufacturer. These characteristics directly influence long-term outcomes like chronic pain, fibrosis, wound healing, and recovery. For instance, heavyweight, small-pore meshes are known to cause more scarring and postoperative discomfort, while lightweight, large-pore meshes generally integrate better with tissue and support smoother healing[3]. Without this information, it is difficult to attribute outcomes to either the technique or the mesh. Finally, although mesh was used in both groups, the authors did not assess complications commonly reported in women after mesh-based pelvic procedures, such as dyspareunia and urinary dysfunction[4]. Additionally, a 2020 meta-analysis found that approximately 9% of male patients experienced pain with sexual activity, and over 5% reported sexual dysfunction following inguinal hernia mesh repair[5]. The absence of such evaluations in a female-specific trial reflects a major gap in outcome reporting.

This trial marks a key step toward advancing hernia care for women. To build on this progress, future studies should adopt rigorous strategies to handle crossover, report mesh details thoroughly, and prioritize validated, sex-specific quality-of-life outcomes. Incorporating these elements will promote more scientifically sound and patient-centered advancements in female hernia care.

TITAN Guidelines: This manuscript complies with TITAN Guidelines, 2025, declaring no use of AI[6].

## Data Availability

Not applicable.

## Research registration unique identifying number (UIN)

Not Applicable for this letter to the editor.

## Peer and provenance statement:

Not commissioned, externally peer reviewed.
